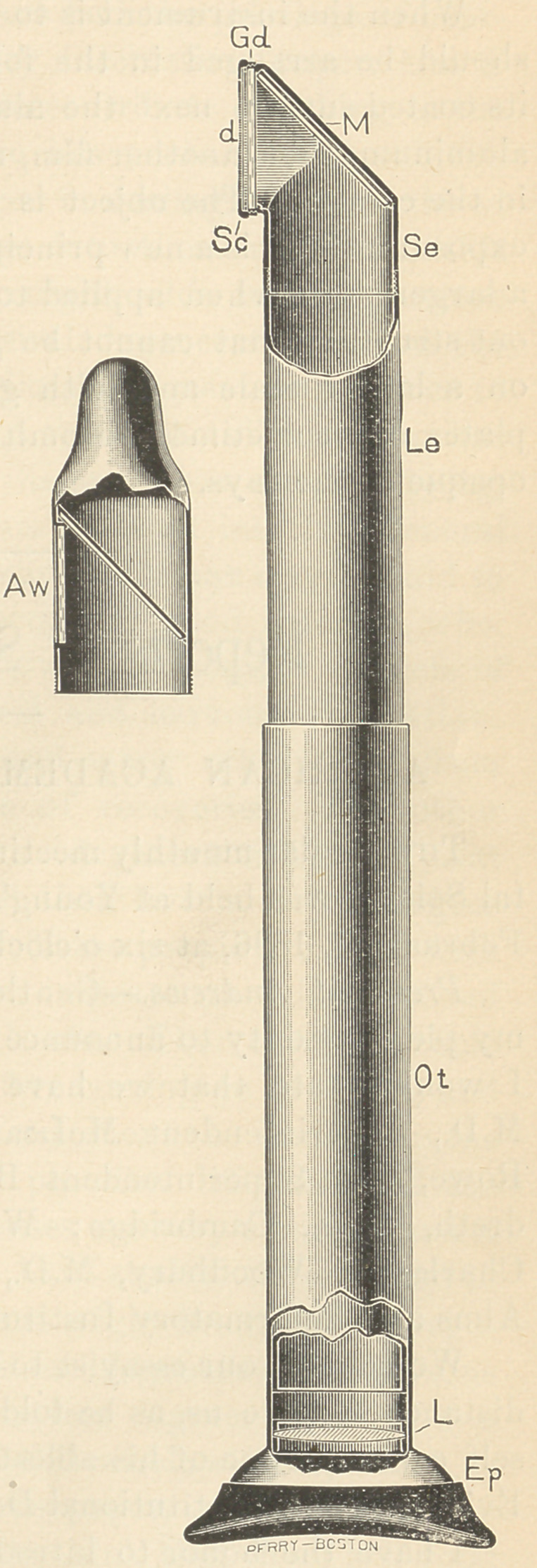# A Roentgen Ray Converter for Internal Use

**Published:** 1896-07

**Authors:** William Rollins

**Affiliations:** Boston, Mass.


### A ROENTGEN RAY CONVERTER FOR INTERNAL USE.

BY WILLIAM ROLLINS, BOSTON, MASS.

Roentgen's discovery that fluorescent substances converted his rays into light rays has been of less value to dentists than to surgeons, for it would stretch even a large mouth to put a Crookes tube or an Edison fluoroscope into it. The small size of the instrument here figured is therefore an advantage.

It consists of a metal tube bent at a right angle. The short end (Se) is closed water- and light-tight by an aluminum disk three one-thousandths of an inch thick. Beneath and separated by a narrow ring is a glass disk (Gd) of the same thickness, with the surface next the aluminum coated with Edison's tungsdate of lime. In the angle is a quartz mirror (M). Over the long end of the tube slides another tube with a flaring end (Ep), having a rim of soft rubber fitting closely about the eye. In the end is a lens (L). By sliding the tube the image formed in the mirror is brought to a focus in the eye.

To use the instrument the Crookes tube is held outside the mouth with its radiant point opposite the aluminum disk, which is pressed against the mucous membrane inside. After use, to sterilize the instrument it is only necessary to remove the mirror and coated glass, when the other parts, which alone come in contact with the tissues, can be baked without injury. The short end (Se) unscrews to allow of the use of other forms. The second here figured is used in examining for stone in the bladder. To photograph with the instrument the film is cut into disks with a punch and placed under the aluminum disk, with a second disk behind it to prevent the light entering the long end from reaching it. The tungsdate of lime plates were coated for me by Dr. F. H. Williams, to whom I am indebted for the use of his powerful apparatus in testing different forms of the instrument.

When the instrument is to be used as a camera alone, the films should be arranged in the following way: Place one film with its coated surface next the aluminum disk; back of this a disk of aluminum, then another film, repeating this until twelve films are in the camera. The object is to give the film varying amounts of exposure. This is a new principle in Roentgen photography, and on a larger scale when applied to other parts of the body will bring out structure that cannot be got in any other way. In working on a larger scale and with glass plates the aluminum dividing plates can sometimes be omitted, as the glass itself is somewhat opaque to the rays.

## Figures and Tables

**Figure f1:**